# Frequencies of 23 Functionally Significant Variant Alleles Related with Metabolism of Antineoplastic Drugs in the Chilean Population: Comparison with Caucasian and Asian Populations

**DOI:** 10.3389/fgene.2012.00229

**Published:** 2012-11-02

**Authors:** Ángela Roco, Luis Quiñones, José A. G. Agúndez, Elena García-Martín, Valentina Squicciarini, Carla Miranda, Joselyn Garay, Nancy Farfán, Iván Saavedra, Dante Cáceres, Carol Ibarra, Nelson Varela

**Affiliations:** ^1^Center of Pharmacological and Toxicological Research (IFT), Molecular and Clinical Pharmacology Program, Instituto de Ciencias Biomédicas, Faculty of Medicine, University of ChileSantiago, Chile; ^2^School of Pharmacy, Faculty of Medicine, Andrés Bello UniversitySantiago, Chile; ^3^San Juan de Dios HospitalSantiago, Chile; ^4^Department of Pharmacology, University of ExtremaduraCáceres, Spain; ^5^Department of Biochemistry, University of ExtremaduraCáceres, Spain; ^6^School of Public Health, Faculty of Medicine, University of ChileSantiago, Chile; ^7^Santo Tomás UniversitySantiago, Chile; ^8^School of Medical Technology, University of ChileSantiago, Chile

**Keywords:** polymorphisms, biomarkers, CYP450, MTHFR, antineoplastic, biotransformation, pharmacogenetics, pharmacogenomics

## Abstract

Cancer is a leading cause of death worldwide. The cancer incidence rate in Chile is 133.7/100,000 inhabitants and it is the second cause of death, after cardiovascular diseases. Most of the antineoplastic drugs are metabolized to be detoxified, and some of them to be activated. Genetic polymorphisms of drug-metabolizing enzymes can induce deep changes in enzyme activity, leading to individual variability in drug efficacy and/or toxicity. The present research describes the presence of genetic polymorphisms in the Chilean population, which might be useful in public health programs for personalized treatment of cancer, and compares these frequencies with those reported for Asian and Caucasian populations, as a contribution to the evaluation of ethnic differences in the response to chemotherapy. We analyzed 23 polymorphisms in a group of 253 unrelated Chilean volunteers from the general population. The results showed that CYP2A6*2, CYP2A6*3, CYP2D6*3, CYP2C19*3, and CYP3A4*17 variant alleles are virtually absent in Chileans. CYP1A1*2A allele frequency (0.37) is similar to that of Caucasians and higher than that reported for Japanese people. Allele frequencies for CYP3A5*3(0.76) and CYP2C9*3(0.04) are similar to those observed in Japanese people. CYP1A1*2C(0.32), CYP1A2*1F(0.77), CYP3A4*1B(0.06), CYP2D6*2(0.41), and *MTHFR* T(0.52) allele frequencies are higher than the observed either in Caucasian or in Japanese populations. Conversely, CYP2C19*2 allelic frequency (0.12), and genotype frequencies for *GSTT1* null (0.11) and *GSTM1* null (0.36) are lower than those observed in both populations. Finally, allele frequencies for CYP2A6*4(0.04), CYP2C8*3(0.06), CYP2C9*2(0.06), CYP2D6*4(0.12), CYP2E1*5B(0.14), CYP2E1*6(0.19), and UGT2B7*2(0.40) are intermediate in relation to those described in Caucasian and in Japanese populations, as expected according to the ethnic origin of the Chilean population. In conclusion, our findings support the idea that ethnic variability must be considered in the pharmacogenomic assessment of cancer pharmacotherapy, especially in mixed populations and for drugs with a narrow safety range.

## Introduction

Cancer is a leading cause of death worldwide and the total number of cases globally is increasing. The number of cancer deaths is projected to increase 45% from 2007 to 2030 (from 7.9 million to 11.5 million deaths), influenced in part by an increasing and aging global population. The estimated rise takes into account expected slight declines in death rates for some cancers in high resource countries. New cases of cancer in the same period are estimated to increase from 11.3 million in 2007 to 15.5 million in 2030 (WHO, 2011). In Chile cancer have a rate of 133.7 × 100,000 inhabitants, is the second cause of death after cardiovascular diseases with a sustained increase in the time both, in the rates and in proportion of deaths. Chile has a number of 30,000 new cases and 36,000 hospitalizations per year. The first cause of cancer death in Chilean population is gastric cancer for both genders, with a rate of 19.8 × 100,000 inhabitants, followed by lung cancer (15 × 100,000 inhabitants), prostate (10.4 × 100,000 inhabitants), and gallbladder (8.1 × 100,000 inhabitants; DEIS, 2011).

Though the principal achievements in the fight against cancer has been those related to preventive measures, the success in the treatment of an established cancer depends on the cancer stage when detected, on the type of cancer, and on its location. It is quite infrequent to find patients considered as “therapeutical success” because there is always a possibility that a tumor process may appear back (Arrastoa, 1998).

Chemotherapy for cancers has progressed from its introduction to clinical practice and constitutes the mainstay modality of therapy in these pathologies. Nevertheless, its use is limited by the inability to predict the response. In most cases the therapy choice is empirical. Nowadays there are more than 100 antineoplastic drugs, which are used either alone or combined. A combined therapy allows that drugs with different mechanism of action work together to destroy the larger possible amount of tumor cells, in order to reduce the possibility of resistance to a particular antineoplastic drug. The therapy to select, the dose, the method of administration and the frequency, and duration of the treatment, will depend on the type of cancer, its location, the rate of growth, how it is affecting the normal functions of the body, as well as on the patient’s general health condition. Usually therapies can be managed by means of cycles that alternate drug administration with washout periods that allow healthy cells to recover from the effect of the medication.

The biotransformation of drugs, including antineoplastic agents, is done basically in two phases: phase I, catalyzed mainly by the cytochrome P450 system and phase II, by transferases that catalyze reactions of conjugation of xenobiotics with diverse molecules of endogenous nature as glucuronic acid, sulfates, acetate, glutathione, or some amino acids. The final outcome of both phases is to increase the hydrophilicity of chemicals facilitating the excretion through urine or bile (Rooney et al., 2004).

There is limited information available in human beings regarding the metabolism and pharmacokinetics interaction of antineoplastic drugs. Nevertheless, it is well known that clinical significant interactions exist between drugs that can render them less efficient if used simultaneously, and in some cases, produce unexpected effects.

The cytochrome P450 (CYP) system is the most important metabolic system responsible for the oxidation of numerous chemotherapeutic agents, and it is responsible to a great extent for the variability observed in response to drugs. For example, some enzymes of the CYP3A family play an important role in the metabolism of epidophilotoxin, ifosfamide, tamoxifen, taxol, and vinca alkaloids. Cytochrome P450 enzymes are also of great importance in the study of chemotherapy resistance. (Kivisto et al., 1995; Yao et al., 2000; Lin and Yu, 2001; García-Martín et al., 2006a; Quiñones et al., 2008).

Genetic polymorphisms of CYP enzymes can produce deep changes in enzyme activity, thus determining the individual response to a certain drug leading to poor, intermediate, extensive, or ultrarapid metabolizer phenotypes (Ingelman-Sundber, 2005; Jin, 2005). On the other hand, the glucuronidation of drugs is carried out by the UDP Glucuronyl transferases (UGT), which are also polymorphic in human beings, adding to the diversity of this reaction. In this respect, UGT2B7 has unique specificity for 3,4-catechol estrogens and estriol, suggesting that it may play an important role in regulating the level and activity of these potent estrogen metabolites. It is also able to conjugate major classes of drugs such as analgesics (morphine), carboxylic non-steroidal anti-inflammatory drugs (ketoprofen), anticarcinogens (all-trans retinoic acid), and tamoxifen. 802C/T mutation leads to UGT2B7 variants UGT2B7*1 (Y268) and UGT2B7*2 (H268) which has been suggested to increase the activity of the enzyme (Ritter et al., 1990; Coffman et al., 1997; Barre et al., 2007; Lazarus et al., 2009). Similarly, the human glutathione-*S*-transferases (GSTs), are polymorphic isoenzymes which show a wide subcellular distribution. GST alpha (α), kappa (κ), mu (μ), pi (π), omega (ω), sigma (σ), theta (θ), and zeta (Z are being studied as possible genetic biomarkers of cancer and its chemotherapeutic treatment (Guengerich et al., 1992; Quiñones et al., 1999; Bredschneider et al., 2002). These enzymes are fundamental in the oxidative processes and detoxification of a wide variety of xenobiotics, including many chemotherapeutic drugs (Massad-Massade et al., 1997; Sargent et al., 1999; Clapper, 2000; Bredschneider et al., 2002; Petros et al., 2005). These polymorphisms have been postulated also as biomarkers for susceptibility to diverse types of cancer (IARC, 1999; Clapper, 2000; Au, 2001; Quiñones et al., 2001; Acevedo et al., 2003; Keshava et al., 2004; Lee et al., 2006; Cordero et al., 2010; Singh et al., 2011) showing marked interethnic differences (Stephens et al., 1994; Muñoz et al., 1998; Garte et al., 2001; Yasuda et al., 2008).

Another relevant enzyme is methylene tetrahydrofolate reductase (MTHFR) which converts 5,10-methylene tetrahydrofolate to 5-methyl tetrahydrofolate. This reaction is required for the multistep process that converts the amino acid homocysteine to another amino acid, methionine. Polymorphisms of this enzyme predispose to serious bone marrow toxicity during treatment with agents that inhibit folate synthesis (e.g., methotrexate; Chiuslo et al., 2002; Yang et al., 2012).

Because of enzymes CYP1A1, CYP1A2, CYP2A6, CYP3A4/5, CYP2C8, CYP2C9, CYP2C19, CYP2E1, CYP2D6, GSTM1, GSTT1, UGT2B7, and MTHFR take part in the metabolism of oncological drugs (Table 1), the main goal of this study was to determine the allele frequencies of variants of these enzymes in a group representative of the Chilean population in order to describe genetic polymorphisms that might be useful in public health programs, and to compare these frequencies with other populations, as the first approximation to the evaluation of ethnic differences in the response to chemotherapies.

**Table 1:** **General characteristics of the studied population**.

Sex	Women	Men	Total
Number	155	98	253
Age (years)*	33.6 ± 13.6	28.7 ± 10.8	31.7 ± 12.8
Weight (kg)*	61.8 ± 9.1	74.9 ± 10.1	66.8 ± 11.4
Height (m)*	1.60 ± 0.06	1.73 ± 0.07	1.65 ± 0.09
BMI (Kgm^−2^)*	24.1 ± 3.3	25.0 ± 2.9	24.5 ± 3.2
A_A–C_: 37%**			

*Information is expressed as averages ± SD; BMI: body mass index.

**Amerindian-Caucasian Admixture (%).

## Materials and Methods

### Study population

Blood samples were obtained from 253 unrelated volunteers living in Santiago of Chile (both sexes, 27–55 years old). The study group has a 37% Amerindian-Caucasian admixture, as determined by ABO blood group distribution (Valenzuela, 1988). The research was authorized by the Ethics Committee of the Faculty of Medicine of the University of Chile. All subjects signed an authorized informed consent. Table 1 shows general characteristics of the studied population.

### DNA extraction

Extraction of genomic DNA was done by a standard procedure from whole blood using a commercial kit (High Pure PCR Template Preparation Kit, Roche Diagnostics^®^) and DNA samples were stored at -20°C until further analysis.

### Genotyping

Genomic DNAs from peripheral blood were amplified by PCR using specific primers for detection of the specific allelic variants in study. For detection of polymorphisms CYP1A1*2A, CYP1A1*2C, CYP1A2*1F, CYP2E1*5B, CYP2E1*6, CYP2A6*2, CYP2A6*3, CYP2A6*4, CYP2D6*2, CYP2C9*2, CYP2C19*2, CYP3A4*1B, CYP3A4*17, CYP3A5*3, and UGT2B7*2, amplicons were surrendered to digestion with the appropriate restriction enzyme to be analyzed through electrophoresis, in agarose gel (2%) or polyacrylamide gel (16%) according to methods previously reported (Hayashi et al., 1999; Kitagawa et al., 1999; Quiñones et al., 1999; Cavalli et al., 2001; LEE et al., 2005; Lin et al., 2005). CYP2D6*3 and CYP2D6*4 were analyzed by means of allele-specific PCR (Heim and Meyer, 1990; Amrithraj et al., 2006). CYP2C9*3, CYP2C19*3, and CYP2C8*3 polymorphisms were analyzed with *Taqman* probes in an ABI 7500 Real Time PCR system, using specific probes previously described (Agúndez et al., 2009). Deletions of *GSTM1* and *GSTT1* were analyzed through PCR using β-globin gene as control (Quiñones et al., 1999; Rebbeck et al., 1999). Heterozygous and homozygous non-null individuals could not be differentiated, therefore double null genotypes (−/−) are the null genotypes reported. Finally, to detect *MTHFR* C677T polymorphism we use a commercial Real Time kit (Roche Diagnostics^®^).

## Results and Discussion

Pharmacogenetic research is directed to identify genes or gene products associated with diseases and, especially, allelic variants in enzymes of biotransformation that alter the individual response to drugs. These variants can modify the magnitude of the pharmacological effect, the threshold of toxicity, the efficacy of the drug, side effects, and drug–drug interactions. In this respect, it is particularly important at the time, to define pharmacogenetic profiles of patients with cancer to determine suitable dosages, to improve efficacy, to avoid adverse reactions of the traditionally used drugs, and to develop new drugs according to the genetic – metabolic profile of the patients (Wilkinson, 2000). Moreover, ethnicity plays an important role in pharmacokinetics and pharmacodynamics of drugs (Ling and Lee, 2011; Kurose et al., 2012) giving rise a more complex situation in “mestizo” populations as South American people. In this sense, Chilean population is a genetic admixture originated primarily between Caucasian (mainly Spaniards) and native-American (mainly Mapuches) from a first single migration of Asians from Siberia 15,000 years ago through Beringia (Reich et al., 2012) and secondly from immigration, mostly from Germany, Croatia, France and Italy. This is a restriction to extrapolate the dosage of drugs with clinical studies performed in other ethnic groups. Another restriction is the poor information about the biotransformation enzyme polymorphisms in Chilean population. According to this, we have studied genetic polymorphisms of several enzymes, in a sub-group of the Chilean population, which metabolize mainly antineoplastic drugs used for chemotherapy in health institutions of Chile (Table 1). General characteristics of the analyzed population are shown in Table 2. The mean age identifies a young adult population which has, in average, a normal mean weight, height, and body mass index and are a representative sample of the middle class Chilean population, which is supported by the Amerindian-Caucasian admixture (37%).

**Table 2:** **Some antineoplastic drugs, substrates of polymorphic enzymes analyzed in this research (http://www.pharmacologyweekly.com/content/pages/cytochrome-cyp-p450-enzyme-medication-herbs-substrates; Quiñones et al., 2008)**.

Drugs	Enzyme	Cancer
Cisplatin	GSTM, GSTT	Breast
Cyclophosphamide	CYP2B6, CYP2C19, CYP3A4	Leukemia, lymphoma
Dacarbazine	CYP1A1, CYP1A2, CYP2E1	Melanoma, sarcoma, lymphoma
Docetaxel	CYP2C8, CYP3A, CYP1B1	Breast, lung, stomach
Doxorubicin	CYP3A4	Sarcoma
Ellipticine	CYP3A4, CYP1A	Leukemia, myeloma, lymphosarcoma
Etoposide	CYP3A4, CYP2E1, CYP1A2	Testicle, lung, breast, leukemia, lymphoma
Ifosfamide	CYP2B6, CYP3A4	Sarcoma, testicle
Imatinib	CYP3A4, CYP3A5	Leukemia
Irinotecan	CYP3A4/5	Colon and rectum
	UGT1A1	
Methotrexate	MTHFR	Leukemia
Mitoxantrone	CYP3A4, CYP1B1	Leukemia, lymphoma
Paclitaxel	CYP2C8, CYP3A4	Breast, lung, ovary
Phortress (2-(4-aminophenyl-benzotiazol)	CYP1A1, CYP1B1	Ovary, breast
Procarbazine	CYP1A1, CYP2B6	Lymphoma, brain, lung, melanoma, testicle
Tamoxifen	CYP3A4, CYP2D6, CYP2C9, CYP2C19, CYP1B1, UGT2B7	Breast
Tegafur	CYP2A6, CYP2C8, CYP1A2	Colon, rectum, stomach
Teniposide	CYP2C19, CYP3A4/5	Leukemia, lung, brain, bladder, myeloma
Thiotepa	CYP2B6, CYP3A4	Bladder
Topotecan	CYP3A4/5	Ovary, lung
Vinblastine	CYP3A4/5	Lymphoma, osteosarcoma
Vincristine	CYP3A4/5	Lymphoma
Vindesine	CYP3A4/5	Lung
Vinrelbine	CYP3A4/5	Lung

The allele and genotype frequencies for metabolic enzymes included in this research are shown in the Table 3. Due to DNA shortage, not all DNA samples were analyzed for all polymorphisms. In Table 4 we compare allele frequencies found in this study in relation to Caucasian and Asian populations.

**Table 3:** **Genotype and allele frequencies of CYP1A1, CYP1A2, CYP2A6, CYP3A4/5, CYP2C8, CYP2C9, CYP2C19, CYP2E1, CYP2D6, UGT2B7, GSTM1, GSTT1 y MTHFR polymorphisms in a Chilean mestizo population**.

CYP1A1*2A	*n*	%	CYP1A1*2C	*n*	%	CYP1A2*1F	*n*	%	CYP2A6*2	*n*	%	CYP2A6*3	*n*	%	CYP2A6*4	*n*	%
*1/*1	112	44.27	*1/*1	79	43.89	*1A/*1A	18	7.11	*1A/*1A	244	100.00	*1A/*1A	253	100.00	*1A/*1A	244	100.00
*1/*2A	95	37.55	*1/*2C	86	47.78	*1A/*1F	81	32.02	*1A/*2	9	0.00	*1A/*3	0	0.00	*1A/*4	0	0.00
*2A/*2A	46	18.18	*2C/*2C	15	8.33	*1F/*1F	154	60.87	*2/*2	0	0.00	*3/*3	0	0.00	*4/*4	9	0.00
TOTAL	253	100.0	TOTAL	180	100.00	TOTAL	253	100.00	TOTAL	253	100.00	TOTAL	253	100.00	TOTAL	253	100.00
fwt	0.63		f*1	0.68		f*1A	0.23		f*1A	0.98		f*1A	1.00		f*1A	0.96	
f*2A	0.37		f*2C	0.32		f*1F	0.77		f*2	0.02		f*3	0.00		f*4	0.04	

CYP2C8*3	***n***	**%**	CYP2C9*2	***n***	**%**	CYP2C9*3	***n***	**%**	CYP2C19*2	***n***	**%**	CYP2C19*3	***n***	**%**	CYP2D6*2	***n***	**%**

*1A/*1A	157	87.22	*1A/*1A	225	88.93	*1A/*1A	167	92.78	*1A/*1A	201	79.45	*1A/*1A	253	100.00	*1A/*1A	88	34.78
*1A/*3	23	12.78	*1A/*2	26	10.28	*1A/*3	13	7.22	*1A/*2	44	17.39	*1A/*3	0	0.00	*1A/*2	121	47.83
*3/*3	0	0.00	*2/*2	2	0.79	*3/*3	0	0.00	*2/*2	8	3.16	*3/*3	0	0.00	*2/*2	44	17.39
TOTAL	180	100.00	TOTAL	253	100.00	TOTAL	180	100.00	TOTAL	253	100.00	TOTAL	253	100.00	TOTAL	253	100.00
f*1A	0.94		f*1A	0.94		f*1A	0.96		f*1A	0.88		f*1A	1.00		f*1A	0.59	
f*3	0.06		f*2	0.06		f*3	0.04		f*2	0.12		f*3	0.00		f*2	0.41	

CYP2D6*3	***n***	**%**	CYP2D6*4	***n***	**%**	CYP2E1*5B	***n***	**%**	CYP2E1*6	***n***	**%**	CYP3A4*1B	***n***	**%**	CYP3A4*17	***n***	**%**

*1A/*1A	248	98.02	*1A/*1A	198	78.26	*1A/*1A	135	75.00	*1A/*1A	123	68.33	*1A/*1A	225	88.93	*1A/*1A	253	100.00
1A/*3	5	1.98	1A/*4	50	19.76	*1A/*5B	39	21.67	*1A/*6	45	25.00	*1A/*1B	28	11.07	*1A/*17	0	0.00
*3/*3	0	0.00	*4/*4	5	1.98	*5B/*5B	6	3.33	*6/*6	12	6.67	*1B/*1B	0	0.00	*17/*17	0	0.00
TOTAL	253	100.00	TOTAL	253	100.00	TOTAL	180	100.00	TOTAL	180	100.00	TOTAL	253	100.00	TOTAL	253	100.00
f*1A	0.99		f*1A	0.88		f*1A	0.86		f*1A	0.81		f*1A	0.95		f*1A	1.00	
f*3	0.01		f*4	0.12		f*5B	0.14		f*6	0.19		*f* *1B	0.06		f*17	0.00	

CYP3A5*3	***n***	**%**	**MTHFR (C677T)**	***n***	**%**	UGT2B7*2	***n***	**%**	**GSTM1**	***n***	**%**	**GSTT1**	***n***	**%**			

*1A/*1A	17	6.72	CC	36	20.00	*1/*1	68	26.88	null	161	63.64	null	160	88.88			
*1A/*3	86	33.99	CT	100	55.56	*1/*2	170	67.19	null	92	36.36	null	20	11.11			
*3/*3	150	59,29	TT	44	20.00	*2/*2	15	5.93	TOTAL	253	100.00	TOTAL	180	100.00			
TOTAL	253	100.00	TOTAL	180	24.44	TOTAL	253	100.00									
f*1A	0.24		fc	0.48		f*1	0.60										
f*3	0.76		ft	0.52		f*2	0.40										

**Table 4:** **Comparison of allelic frequencies of CYP1A1, CYP1A2, CYP2A6, CYP3A4, CYP3A5, CYP2C8, CYP2C9, CYP2C19, CYP2D6, CYP2E1, GSTM1, GSTT1, UGT2B7, and MTHFR in Caucasian, Japanese, and Chilean populations**.

					Alle Frequencies			
Enzyme	Allele	rs	Gene change(protein change)	Enzyme activity	Chilean	Caucasian	Japanese	Reference
CYP1A1	*2A	rs4646903	T3801C	Increased	0.37	0.36	0.09	Murata et al. (2001)
	*2C	rs1048943	A2454G (I462V)	Decreased	0.32	0.22	0.05	Murata et al. (2001)
CYP1A2	*1F	rs762551	C-163A	Higher inducibility	0.77	0.68	0.61	Sachse et al. (1999), Skarke et al. (2005), Chida et al. (1999)
CYP2A6	*2	rs1801272	T479A (L160H)	None	0.02	0.02	0.00	Nakajima et al. (2006), Chen et al. (1999)
	*3	rs56256500	CYP2A6/CYP2A7 hybrid	Decreased?	0.00	0.00	0.00	
	*4	rs3892097	Deletion	None	0.04	0.00	0.19	
CYP3A4	*1B	rs2740574	A-392G	Decreased	0.06	0.04	0.00	Paris et al. (1999)
	*17	rs4987161	T15615C	Decreased	0.00	0.00	0.00	
CYP3A5	*3	rs776746	A6986G Splicing defect	Decreased	0.76	0.70	0.75	Kurose et al. (2012), Roy et al. (2005)
CYP2C8	*3	rs10509681	G2130A, A30411G (R139K, K399R)	Decreased	0.06	0.16	0.00	Agúndez et al. (2009)
CYP2C9	*2	rs1799853	C3608T (R144C)	Decreased	0.06	0.08	0.00	Agúndez et al. (2009), Sullivan-Klose et al. (1996), Nasu et al. (1997)
	*3	rs1057910	A42614C (I359L)	Decreased	0.04	0.06	0.03	
CYP2C19	*2	rs28399504	G19154A Splicing defect	None	0.12	0.14	0.23	Kurose et al. (2012)
	*3	rs4986893	G17948A W212X	None	0.00	0.00	0.11	
CYP2D6	*2	rs16947	C2850T (R296C)	Normal	0.41	0.32	0.13	Kurose et al. (2012)
	*3	rs35742686	del2549A(259 Frame shift)	None	0.01	0.02	0.00	
	*4	rs3892097	G1846A Splicing defect	None	0.12	0.21	0.00	
CYP2E1	*5B	rs2031920/rs3813867	G-1293C/C-1053T	Decreased	0.15	0.04	0.20	Krishnakumar et al. (2012)
	*6	rs6413432	T7632A	Decreased	0.22	0.08	0.29	Krishnakumar et al. (2012)
GSTM1	null	SNP500Cancer ID – GSTM1-02	Null deletion	None	0.20	0.45	0.55	Kurose et al. (2012)
GSTT1	null	SNP500Cancer ID – GSTT1-02	Null Deletion	None	0.11	0.52	0.20	Kurose et al. (2012)
UGT2B7	*2	rs7439366	C802T (H268Y)	Decreased	0.40	0.49	0.27	Bhasker et al. (2000)
MTHFR	T	rs1801133	C677T (A222V)	Thermolabile enzyme	0.52	0.41	0.32	Matsuo et al. (2001), Chen et al. (1998)

Our results show the absence of variant alleles CYP2A6*3, CYP2C19*3, and CYP3A4*17 such as it was also observed for Caucasians. In Japanese people CYP2A6*3 and CYP3A4*17 are also absent, but the CYP2C19*3 allele has a frequency of 0.11. CYP1A1*2A and CYP2A6*2 allele frequencies are similar to Caucasians but higher than the reported for Japanese people. CYP3A5*3 and CYP2C9*3 frequencies are similar to those in Japanese people, but different to the Caucasian people. CYP1A1*2C, CYP1A2*1F, CYP3A4*1B, and CYP2D6*2 allelic frequencies are higher than those observed either in Caucasian or Japanese populations. *MTHFR*T allele frequency is higher than the observed in Caucasian and Japanese population, but also than the frequency reported previously by Nitsche et al. (2003) in other group of the Chilean population. We suggest that this difference could be explained by different genetic composition of the previously studied group, which could be more similar to Caucasians. Unfortunately, Nitsche et al. no reported the Amerindian-Caucasian admixture percentage to evaluate this point.

On the other hand, CYP2C19*2, *GSTT1* null and *GSTM1* null frequencies are lower than those reported in Caucasian or Japanese population. Finally for CYP2C8*3, CYP2C9*2 and *3, CYP2D6*3, CYP2D6*4, CYP2E1*5B and *6, as well as for UGT2B7*2 the frequencies described for Chileans are intermediate in relation to those described for Caucasian and Japanese population (Sullivan-Klose et al., 1996; Nasu et al., 1997; Chen et al., 1998, 1999; Chida et al., 1999; Paris et al., 1999; Sachse et al., 1999; Bhasker et al., 2000; Matsuo et al., 2001; Murata et al., 2001; Roy et al., 2005; Skarke et al., 2005; Nakajima et al., 2006; Krishnakumar et al., 2012; Kurose et al., 2012), which is expected because Chilean population is considered a mixed ethnicity between both races.

Some limitations in this study should be pointed out. Only some polymorphism of the many (>80) CYP2D6 known were genotyped. We select only CYP2D6 polymorphisms that have shown better reported relationship with plasma levels of antineoplastic drugs and those that have higher allele frequencies, based on a previous pilot study in Chilean subjects (Dr. Monica Acuña, Personal Communication). Similarly, same criteria were used for choosing the other studied polymorphisms, based on both literature and our own previous research. Nevertheless, some potentially relevant CYP variants are currently under investigation in our laboratory (CYP2D6*2xN, CYP2D6*5, CYP2D6*10, CYP2C8*3, and CYP2C8*4), to complement the results shown in this paper.

Additionally, we have no data for other relevant polymorphic enzymes, such as for example, CYP1B1 and CYP2B6, responsible for the metabolism of several antineoplastic drugs (Quiñones et al., 2008) and UDP Glucuronyl transferase 1A1, involved in metabolism of irinotecan (Dias et al., 2012). We did not analyze thiopurine *S*-methyltransferase (TPMT), a cytosolic phase II enzyme involved in the metabolism of azathioprine, thiopurine, and thioguanine (Zhou, 2006). However the frequency of four allelic variants of this gene (*2, *3A, *3B, and *3C) were analyzed previously in Chilean population by Alvarez et al. (2009) showing that the presence of *3A allele is the most prevalent, which is similar to Caucasians, giving a first approach to the use of this polymorphism in clinical practice in Chilean patients.

Another limitation of the present research is the use of Japanese population as proxy of the ancestral Asians of Chilean people. We use this population as reference due to two main facts: (a) there is very good and complete information about these polymorphisms in Japanese people and (b) recently, have been reported no great differences among Japanese and other Asian populations, particularly with respect to Chinese population (Kurose et al., 2012).

On the other hand, drug–drug pharmacological interactions, some epigenetic and environmental factors, and alternative metabolic routes should not be excluded to describe response to antineoplastic agents, which is *per se* a multifactorial event. Thus, the research in this area must identify these factors and potential gene – environment interactions that modulate response to these drugs.

Some polymorphisms have been studied in other South American countries (Isaza et al., 2000; Gaspar et al., 2002; Fernández et al., 2004; Gattas et al., 2004; Vianna-Jorge et al., 2004; Almeida et al., 2005; Lizcano Fernández, 2005; Rossini et al., 2006; Schlawicke et al., 2007; Canalle et al., 2008; Rodríguez et al., 2008; Castaño-Molina et al., 2009; Dorado et al., 2011), but the comparison with Chilean Mestizo population is very difficult because of the divergent origin of these populations. Chilean population is different from other South American countries, from Brazilian people for example, which have African and Portuguese contribution with great pharmacogenomic diversity, or from Argentina and Uruguay populations, which are multiethnic countries, with Amerindian-European admixture, but mostly with Italians (Lizcano Fernández, 2005). In this respect, in South America, the region one of the most diverse genetic background in the world, four main components have contributed to the present-day population: Amerindians (pre-Columbian inhabitants); Iberians (conquerors) who dominated the continent until the nineteenth century, Africans (imported as slaves by the colonizers); and post-independence immigrants from overseas (mostly Italy and Germany but also from France, South Asia, and Japan). Therefore, we suggest that studies of these pharmacogenes in Chileans should be used to develop pharmacogenetic tools for this specific population, rather than extrapolating results obtained to other populations.

The frequencies observed in metabolic polymorphism studied in Chilean population were distinct from paternal races. These results contribute to better understanding of the basis of ethnic variation in drug metabolism and response (Agúndez, 2004; García-Martín et al., 2006b; Yasuda et al., 2008), and suggest a complex genetic profile of this “mestizo” population, which should be considered in pharmacotherapy, especially for drugs with a narrow safety range, particularly in cancer chemotherapy (García-Martín, 2008; Quiñones et al., 2008). These established genotype frequencies may be used for studying the phenotype variation in further studies. Thus, it may be a good contribution for further studies on the clinical application of pharmacogenomics in Asian-Caucasian mixed races.

## Conclusion

Profound variation in polymorphisms of metabolizing enzymes have been described in diverse populations, including enzymes that take part in the metabolism of chemotherapeutics drugs (50). In this sense, our results agree with these observations when we compare the analyzed sub-group with Asian and Caucasian populations (Table 4).

As Chilean population represents a mixed ethnicity mainly between native-Americans and Caucasians (mostly Spaniards) the data obtained might help to understand inter ethnic differences not only for single polymorphisms, but also the function of simultaneous polymorphisms in metabolic genes in each subject, to explain differences in response to chemotherapy.

This investigation contributes to have a first pattern of several relevant polymorphisms in metabolizing enzymes (*CYP1A1*, *CYP1A2*, *CYP2A6*, *CYP2C8*, *CYP2C9*, *CYP2C19*, *CYP2D6*, *CYP2E1*, *CYP3A4/5*, *GSTM1*, *GSTT1*, *UGT2B7*, *and MTHFR*) in Chilean people, which can give course for a genetic – population investigation of these polymorphisms helping to the understanding of susceptibility to drugs and pathologies in this population, which already has been suggested by our group of research for some specific genes (Quiñones et al., 1999, 2001, 2008; Acevedo et al., 2003; Lee et al., 2006; Cordero et al., 2010).

The knowledge of genetic variants involved in the metabolism of the antineoplastic drugs in the Chilean population will help to the prediction of their clinical efficacy and/or toxicity, and therefore, will help us to the design personalized cancer treatments to improve therapeutic response and diminish the adverse effects improving cost-efficacy of treatments.

Finally, based on scientific literature and our experience, we believe that, at least in Chilean population, MTHFR/methotrexate, GST/cisplatin, and CYP2D6/tamoxifen are the potential more relevant gene/drug pairs which are closer for monitoring use in clinical practice.

## Conflict of Interest Statement

The authors declare that the research was conducted in the absence of any commercial or financial relationships that could be construed as a potential conflict of interest.
